# Supplementary material for: Antibiotics modulate activity of mouse and human dorsal root ganglia neurons
Source: bioRxiv. 2026 Sep 16:2026.09.10.750724. Preprint. [Version 1] doi: 10.64898/2026.09.10.750724 (PMC13596543; doi:10.64898/2026.09.10.750724)
Supplement: Supplementary file 1 [file media-1.pdf]

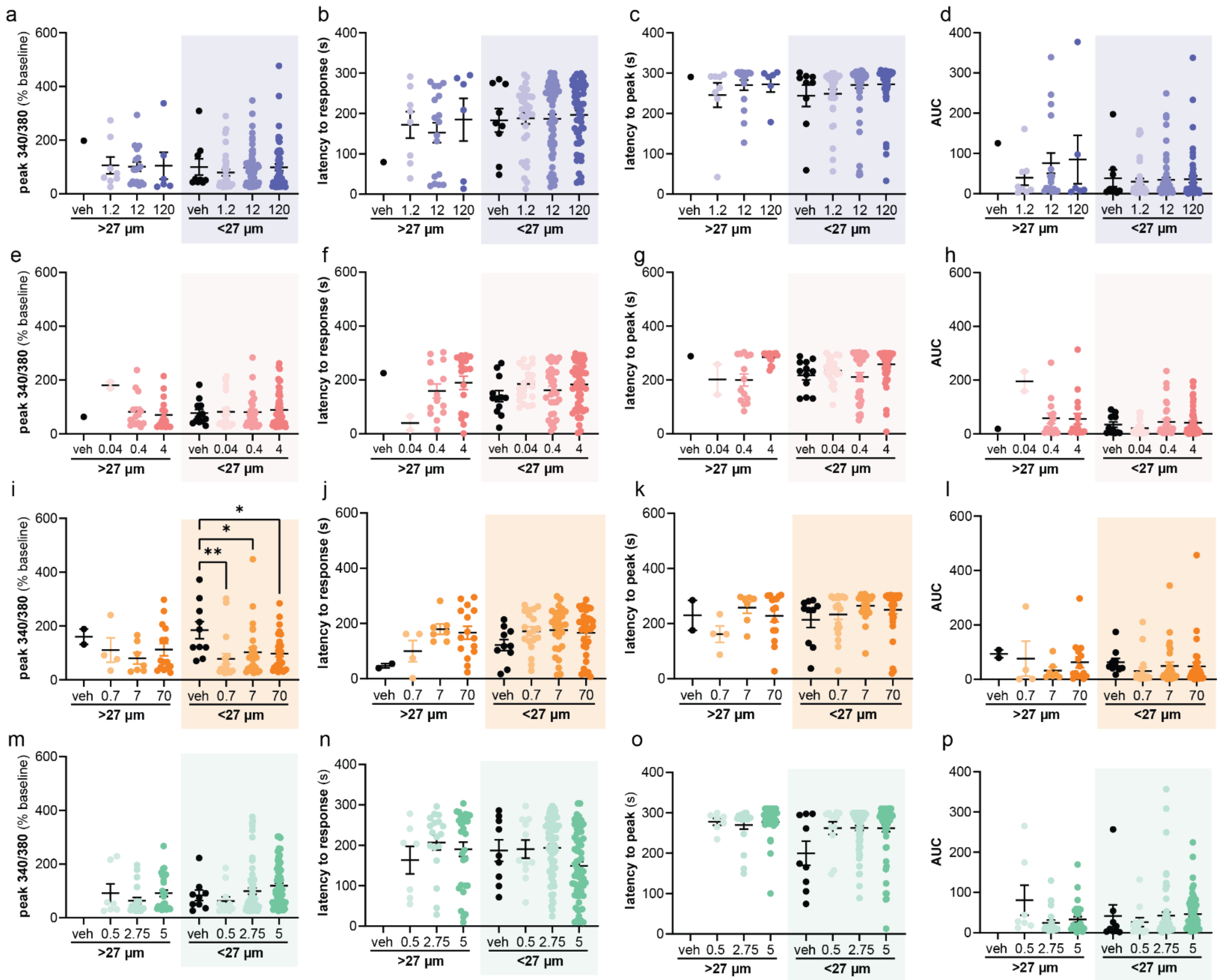

Figure S1. Antibiotics do not change magnitude of response or response kinetics in mDRG neurons *in vitro*. Comparison of antibiotic response peak magnitude, response latency to drug, peak response time, and area under the curve of **a.-d.**, cephalexin (1.2, 12, 120 µg/mL), **e.-h.**, azithromycin (0.04, 0.4, 4 µg/mL), **i.-l.**, amoxicillin (0.7, 7, 70 µg/mL), or **m.-p.**, doxycycline (0.5, 2.75, 5 µg/mL). Large (>27 µm) and small diameter soma (≤27 µm) were analyzed independently. Numbers on top of or within bars indicate responsive neurons/total neurons analyzed; n=4 mice per drug. Statistical comparisons versus vehicle were performed using two-tailed Fisher's exact tests within each size group; *P< 0.05, **P < 0.01, ***P < 0.001, **** P< 0.0001.

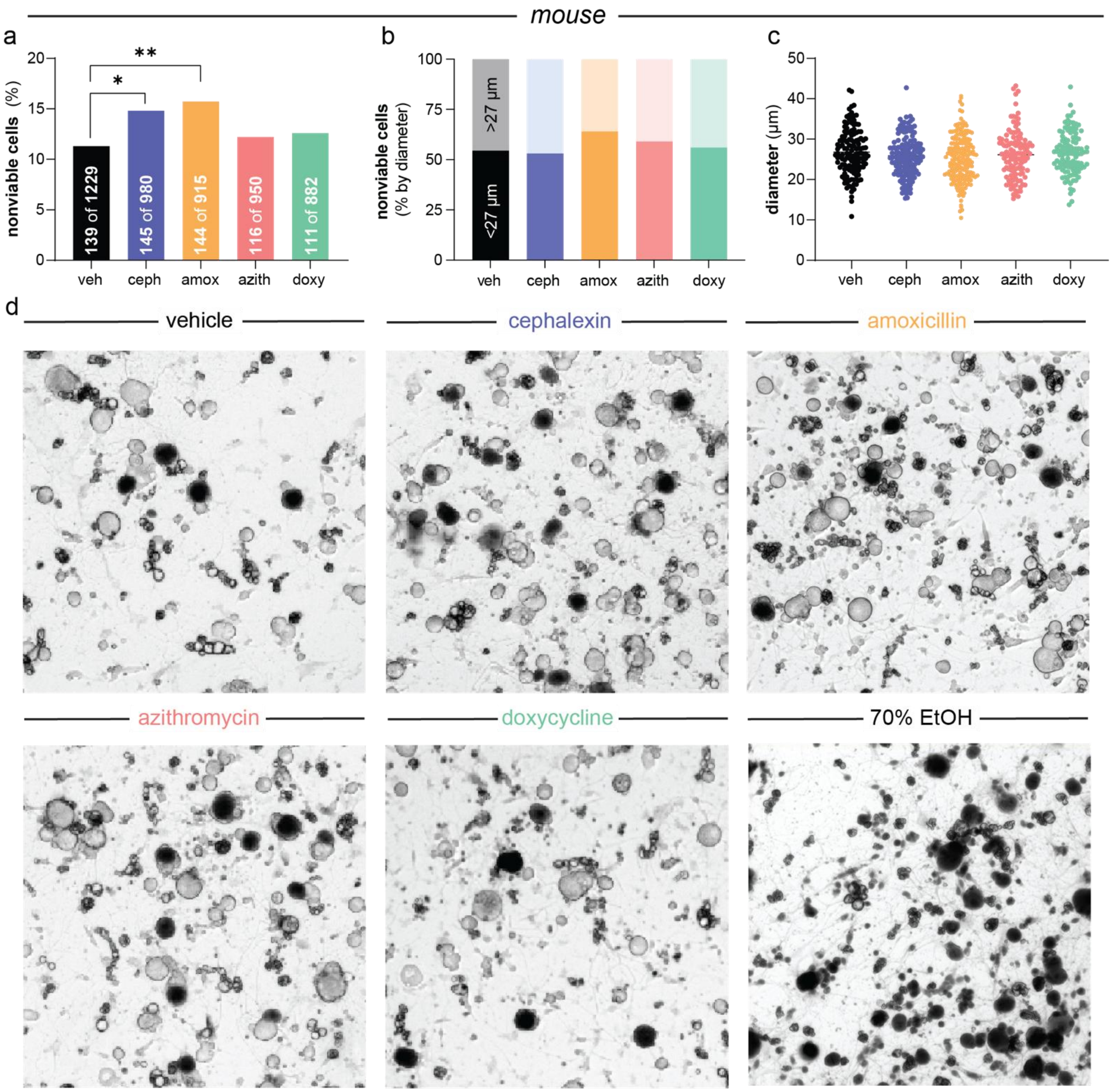

Figure S2. Cephalexin and amoxicillin induce modest cell death in mouse DRG neurons *in vitro*. a., Quantification of nonviable cells after 5-minute exposure to antibiotics or control. **b.**, Percentage of nonviable cells separated large (>27 µm) and small diameter soma (≤27 µm). cephalexin (12 µg/mL), **c**., diameter of all the cells across antibiotic drugs. **d.**, representative images of mDRG neurons exposed to vehicle, cephalexin (12 µg/mL), amoxicillin (7 µg/mL), azithromycin (0.4 µg/mL), doxycycline, and 70% ethanol (positive control). azithromycin or **e.**, doxycycline (5 µg/mL). Statistical comparisons versus vehicle were performed using two-tailed Fisher's exact tests; *P< 0.05, **P < 0.01, ***P < 0.001, **** P< 0.0001.

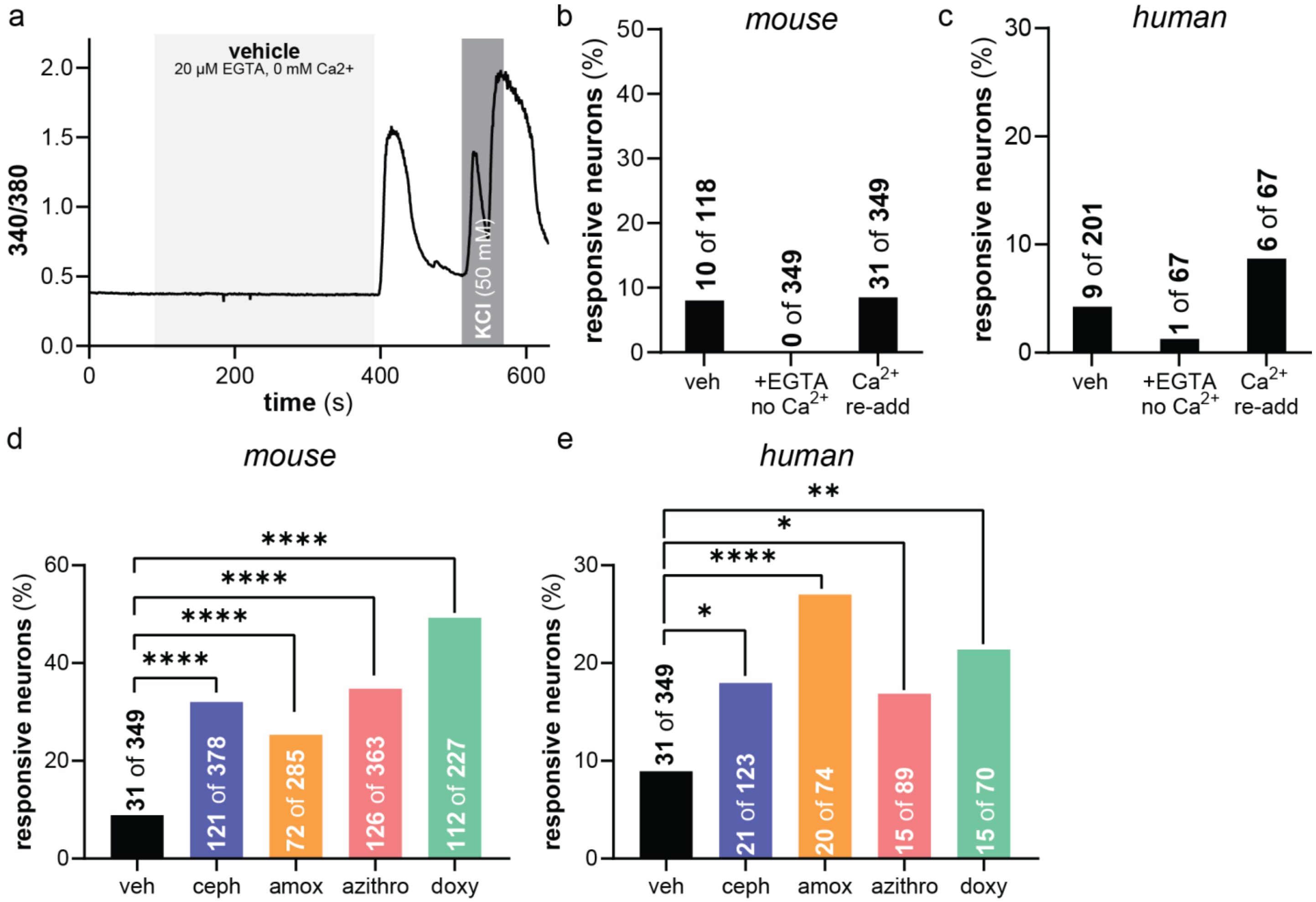

Figure S3. Re-addition of calcium to vehicle exposed mDRG and hDRG neurons does not produce robust changes in the number of responsive neurons. a., Representative calcium transients from mDRG neurons exposed to vehicle. Quantification of **b.**, mDRG and **c.,** hDRG neurons that responded during each phase of imaging (veh alone, +EGTA/0 Ca^{2+}, and Ca^{2+} re-addition). Quantification of **d.,** mDRG and **e.,** hDRG neurons that respond to antibiotics during Ca2+ re-addition compared to vehicle. Statistical comparisons were performed using two-tailed Fisher's exact tests; *P<0.05, **P<0.01, ***P<0.001, ****P<0.0001.

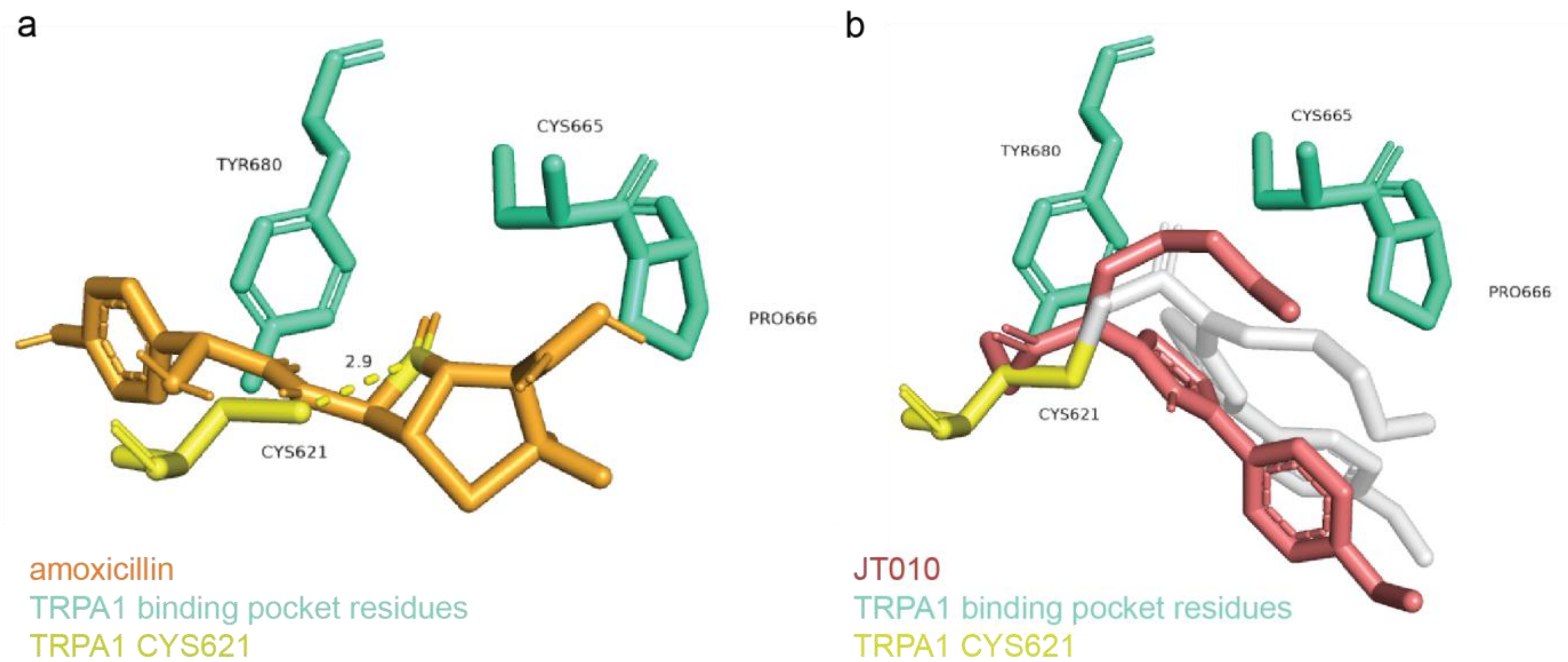

Figure S4. Comparative docking of JT010 and amoxicillin with TRPA1. Predicted docking poses of **a.,** the established TRPA1 agonist JT010 and **b.,** amoxicillin within TRPA1 (PDB: 6PQO), with Cys621 modeled as a flexible residue. Distances between each ligand and Cys621 are indicated.

UTD ID	Age	Sex	COD	DRG Used	DIV	Experiment
UTD-DN0405	19	M	Anoxia/Drug Intoxication	T12, L4 (2X)	3	Amox, Ceph, ENH
UTD-DN0410	35	M	Anoxia/Drug Intoxication	T11, T12, L5	4	Azithro, Dox, ENH
UTD-DN0423	35	M	Head Trauma/Blunt Injury/MVA	L3 (2X), L4 (2X)	3	Amox, Dox, ENH
UTD-DN0426	33	F	Anoxia/Asphyxiation	L5 (2X)	6	Azithro, Ceph, ENH
UTD-DN0429	48	M	CVA/Stroke	L4, L5	4	All
UTD-DN0464	33	M	Anoxia/Asphyxiation/Suicide	L3 (2X)	3	Amox, Azithro, Dox, Ceph (EGTA)
UTD-DN0466	27	M	Head Trauma/Blunt Injury/MVA	T12, L4	6	Amox, Azithro, Dox, Ceph (EGTA)
UTD-DN0473	33	F	CVA/Stroke	L3 (2X)	4	Amox, Azithro, Dox, Ceph, ENH (EGTA)
UTD-DN0493	54	M	CVA/Stroke	L1, L4	3	Dox, Ceph, Dox + RR, Ceph + RR
UTD-DN0495	26	F	Head Trauma	T7, T11, T12, L3	5	Amox, Dox, Dox + RR, Amox + RR
UTD-DN0498	48	M	Head Trauma	L1	5	Azithro, Azithro + RR
UTD-DN0515	48	F	Anoxia	L4 (2X)	4	Azithro + RR
UTD-DN0522	42	F	Cardiac Arrest	T11, L2, L3, L4	3	Amox, Amox + RR
UTD-DN0527	46	M	Anoxia	T12, L2 (2X), L3, L4	5	ENH + EGTA, Azithro + RR, Ceph + RR

Table S1. Organ donor demographics and experimental details for human dorsal root ganglion calcium imaging. Donor information includes age, sex, cause of death (COD), dorsal root ganglion (DRG) level, days in vitro (DIV) at the time of calcium imaging, and experimental conditions tested. M, male; F, female; Amox, amoxicillin; Ceph, cephalexin; Dox, doxycycline; Azithro, azithromycin; ENH, bath solution; RR, ruthenium red.

Ligand	Docking Score
**Cephalexin**	-7.024
**Amoxicillin**	-7.582
**JT010**	-5.872

Table S2. Docking analysis of cephalexin, amoxicillin, and JT010 with TRPA1. Docking was performed using the TRPA1 structure (PDB: 6PQO) with Cys621 modeled as a flexible residue. Docking scores and predicted distances between each ligand and Cys621 are shown. More negative docking scores indicate more favorable predicted binding. JT010, a known covalent ligand of Cys621, was included as a reference.
